# Characteristics of residential areas and transportational walking among frail and non-frail Dutch elderly: does the size of the area matter?

**DOI:** 10.1186/1476-072X-13-7

**Published:** 2014-03-04

**Authors:** Astrid Etman, Carlijn BM Kamphuis, Richard G Prins, Alex Burdorf, Frank H Pierik, Frank J van Lenthe

**Affiliations:** 1 Department of Public Health, Erasmus University MC, P.O. Box 2040, Rotterdam, CA 3000, The Netherlands; 2 Department of Urban Environment and Safety, TNO, P.O. Box 80015, Utrecht, TA 3508, The Netherlands

**Keywords:** Physical environment, Neighbourhood, Elderly, Walking, Transport-related PA

## Abstract

**Background:**

A residential area supportive for walking may facilitate elderly to live longer independently. However, current evidence on area characteristics potentially important for walking among older persons is mixed. This study hypothesized that the importance of area characteristics for transportational walking depends on the size of the area characteristics measured, and older person’s frailty level.

**Methods:**

The study population consisted of 408 Dutch community-dwelling persons aged 65 years and older participating in the Elderly And their Neighborhood (ELANE) study in 2011–2012. Characteristics (aesthetics, functional features, safety, and destinations) of areas surrounding participants’ residences ranging from a buffer of 400 meters up to 1600 meters (based on walking path networks) were linked with self-reported transportational walking using linear regression analyses. In addition, interaction effects between frailty level and area characteristics were tested.

**Results:**

An increase in functional features (e.g. presence of sidewalks and benches) within a 400 meter buffer, in aesthetics (e.g. absence of litter and graffiti) within 800 and 1200 meter buffers, and an increase of one destination per buffer of 400 and 800 meters were associated with more transportational walking, up to 2.89 minutes per two weeks (CI 1.07-7.32; p < 0.05). No differences were found between frail and non-frail elderly.

**Conclusions:**

Better functional and aesthetic features, and more destinations in the residential area of community-dwelling older persons were associated with more transportational walking. The importance of area characteristics for transportational walking differs by area size, but not by frailty level. Neighbourhood improvements may increase transportational walking among older persons, thereby contributing to living longer independently.

## Introduction

In aging populations, the demand for and costs of institutionalised care may become unsustainable in many Western countries. Interestingly, policies aimed at limiting institutionalised care may be in line with the desire of many elderly to live longer independently [1]. Living longer independently, requires a good functional health and it is for this reason that health promotion among elderly becomes increasingly important. Regular physical activity (PA), such as walking, may help to minimize the burden on health care and social care by extending years of active independent living, reducing disability, and improving quality of life, and may increase life expectancy with several years [2,3]. Since up to 83% of the elderly worldwide do not meet recommendations for PA to obtain health benefits [2], PA promotion in this population should be an important part of preventive strategies. Improving transportational walking, such as walking to a shop, seems an excellent strategy since two third of all walks of the elderly are for transportational purposes [4], and elderly can easily make it part of their daily life.

It is increasingly recognized that living longer independently can be facilitated if the residential area around older people’s residences facilitates and inspires elderly to walk for daily activities. There is an increased interest in investigating the role of residential area characteristics (e.g. presence of sidewalks), aesthetics (e.g. presence of trees, absence of graffiti), the presence of facilities (e.g. shops and parks), and safety [5], however studies showed mixed findings concerning the elderly [6]. Methodological shortcomings of current studies are often mentioned as one potential explanation for the inconsistencies, including the use of inappropriate geographical units [7,8]. Commonly used geographical units defined as a one-size predefined area around a person’s residence may not capture sufficient variation for all environmental characteristics [9]. While very common characteristics (e.g. trees) can vary in small areas, larger areas are needed to capture variation in less common characteristics (e.g. parks). Therefore, it was suggested to investigate residential areas of different sizes for the interplay between the physical environment and PA [10]. As older adults are generally less functionally fit than their younger peers, they may use a smaller area around their residences. In addition, the ability to walk may differ between elderly. Frail elderly, being at increased risk of dependent living [11,12], are likely to be bound to smaller areas around their house since they are characterized by lower levels of PA [13,14]. As a consequence, associations between area characteristics and walking may differ by frailty status.

This study aims at investigating whether the association between area characteristics and transportational walking depends on the size of the area for which environmental characteristics are considered, and on the frailty level of the elderly.

## Results

No significant differences in sex and age were found between participants and non-participants. Frail persons were significantly older and more often women. The average total walking time and average time per walk in the past two weeks were both lower for frail persons as compared to non-frail persons (p < 0.001; Table 1).

**Table 1:** Demographics and area characteristics of 408 residents from Spijkenisse within four buffer zones by frailty level

**Buffer size**		**Total (N = 408)**	**Non-frail (N = 307)**	**Frail (N = 101)**
	Sex (% female)	52.9	45.9	74.3**
		Mean ± sd	Mean ± sd	Mean ± sd
	Age (years; range 65–94)	75.1 ± 6.6	73.7 ± 5.7	79.4 ± 7.3**
	Total walking time in last 2 weeks (in minutes)	389.9 ± 579.0	446.2 ± 634.6	218.8 ± 305.0**
	Average total walking time per transportational walk (in minutes)	35.3 ± 32.1	38.8 ± 33.7	24.7 ± 23.9**
400 m	Number of observed streets	39 ± 14	38 ± 14	39 ± 14
	Aesthetics (range 0–22)	11.9 ± 0.9	11.9 ± 0.9	11.9 ± 0.9
	Functional features (range 0–14)	4.8 ± 1.5	4.7 ± 1.5	5.2 ± 1.5*
	Safety (range 0–16)	6.1 ± 1.0	6.1 ± 1.0	6.1 ± 1.0
	Destinations (range 0-∞)	9.4 ± 8.9	8.6 ± 11.9	11.9 ± 10.9*
800 m	Number of observed streets	133 ± 42	132 ± 42	136 ± 43
	Aesthetics (range 0–22)	11.8 ± 0.7	11.8 ± 0.7	11.8 ± 0.7
	Functional features (range 0–14)	4.5 ± 1.0	4.4 ± 1.0	4.7 ± 1.0*
	Safety (range 0–16)	5.9 ± 0.8	5.9 ± 0.8	6.0 ± 0.7
	Destinations (range 0-∞)	26.7 ± 16.7	25.2 ± 16.0	30.9 ± 18.1*
1200 m	Number of observed streets	274 ± 90	273 ± 91	276 ± 87
	Aesthetics (range 0–22)	11.7 ± 0.6	11.7 ± 0.6	11.8 ± 0.7
	Functional features (range 0–14)	4.4 ± 0.8	4.4 ± 0.8	4.5 ± 0.8
	Safety (range 0–16)	5.9 ± 0.7	5.9 ± 0.7	5.9 ± 0.7
	Destinations (range 0-∞)	51.0 ± 24.8	49.5 ± 24.2	55.6 ± 26.1*
1600 m	Number of observed streets	454 ± 147	453 ± 149	457 ± 143
	Aesthetics (range 0–22)	11.7 ± 0.5	11.7 ± 0.5	11.7 ± 0.5
	Functional features (range 0–14)	4.3 ± 0.6	4.3 ± 0.7	4.4 ± 0.6
	Safety (range 0–16)	5.8 ± 0.5	5.8 ± 0.5	5.9 ± 0.5
	Destinations (range 0-∞)	82.1 ± 32.4	80.5 ± 32.6	87.0 ± 31.4

**p < 0.001; *p < 0.05 (=significant higher score as compared to non-frail participants).

Table 1 shows the scores for area characteristics per street for each buffer size. The average scores for aesthetics, functional features, and safety decreased slightly with increasing buffer size; clearly, the accumulated number of destinations within a buffer increased with increasing buffer size. Frail persons had more destinations in their residential area within a buffer up to 1200 meters, and more functional features within a buffer up to 800 meters compared to non-frail persons (Table 1). Aesthetics, functional features, safety, and destinations were all positively correlated with each other, except for the correlation between the number of destinations and aesthetics. Aesthetics and safety showed the highest correlation with a Pearson correlation ranging from 0.72 in the 400 meter buffer to 0.90 in 1600 meter buffer (p < 0.01).

As reported in Table 2, an increase in the aesthetics score of 1 point within 800 and 1200 meter buffers, was found to increase transportational walking with respectively 2.36 and 2.89 minutes per two weeks. The magnitude of the association between functional features and transportational walking was similar across buffer sizes, but was only significant in the 400 meter buffer. An increase of one functional feature per street within 400 meters was associated with 0.72 minutes more walking in 2 weeks. Although safety seemed to be most important in the 400 meter buffer, this association was not found to be significant. An increase of one destination per buffer within 400 and 800 meters was associated with an increase of respectively 1.05 and 1.03 minutes transportational walking per two weeks. The variance in walking time as explained by the models as presented in Table 2, ranged from 6.3% in the 1600 meter buffer up to 8.8% in the 400 meter buffer. No interaction effect of frailty level and area characteristics was found for any of the buffer sizes.

**Table 2:** Associations between area characteristics and transportational walking in four buffer zones (N = 408)

	**400 meters**			**800 meters**			**1200 meters**			**1600 meters**		
**Characteristic**^**a**^	**B**	**(95% CI)**	**p**	**B**	**(95% CI)**	**p**	**B**	**(95% CI)**	**p**	**B**	**(95% CI)**	**p**
Aesthetics	1.24	(0.84-1.85)	0.28	2.36	(1.14-4.90)	0.02	2.89	(1.07-7.32)	0.03	2.16	(0.54-8.51)	0.27
Functional features	0.72	(0.57-0.91)	0.01	0.68	(0.44-1.03)	0.07	0.55	(0.30-1.06)	0.07	0.80	(0.29-1.74)	0.44
Safety	1.18	(0.84-1.67)	0.33	0.70	(0.39-1.26)	0.27	0.82	(0.40-1.76)	0.61	0.80	(0.31-2.02)	0.64
Destinations	1.05	(1.02-1.08)	0.00	1.03	(1.01-1.04)	0.01	1.00	(0.99-1.02)	0.51	1.00	(0.99-1.01)	0.77

^a^Adjustments were made for age, sex, and frailty.

## Discussion

Destinations, functional features, and aesthetics of residential areas were associated with more transportational walking among community-dwelling older persons. An increase of one functional feature per street within a 400 meter buffer surrounding one’s residence, an increase of one destination within 400–800 meter buffers, and an increase in aesthetics within 800–1200 meter buffers were associated with increases in transportational walking up to 2.89 minutes per two weeks.

Higher scores on aesthetics were associated with more time spent on transportational walking, which is in contrast to previous studies [15,16]. This discrepancy may be due to the fact that within these studies aesthetics were measured differently, i.e. by less items or via self-reports. There is inconsistent evidence for associations between the area characteristics functional features and safety and walking [6,17]. The inconsistent findings concerning the association between safety and transportational walking among older persons has been attributed to the complexity of measuring safety [6]. Our measure included both traffic- and social safety indicators, and additional analyses showed that both sets of indicators were not associated with transportational walking in any of the buffers. The association between the presence of destinations and transportational walking was found for buffers up to 800 meters, but was absent in the 1200 and 1600 meter buffers. This finding is in line with studies reporting associations in buffers up to 1000 meters [17,18].

Whereas other studies often use a predefined buffer size [9], our results revealed that associations between area characteristics and walking behaviour differed by buffer size. Nagel et al. found that associations between environmental factors and total walking time among older persons aged 65 years and older were similar across buffer sizes (400 and 800 meters) [17]. We extended this finding, as we also included buffer sizes larger than 800 meters for which also significant associations were found.

A decrease in variation with increasing buffer size was found. This has most likely not biased our results, since we observed associations for aesthetics within 800 and 1200 meters (with lower variation than within 400 meters). It is expected that the municipality of Spijkenisse focussed most on the maintenance and improvement of the (close by) areas where most residents live. This would result in lower scores for area characteristics further away from the residences, and lower average scores when larger buffers are considered. It is unclear how it would affect our results when the mean scores on the area characteristics had remained stable or even increased with increasing buffer sizes, since it depends on the walking behaviour of the elderly and whether they would be able and willing to walk further distances.

A possible explanation for the finding that destinations and functional features were particularly important for transportational walking in small buffer sizes may be that older persons are generally less functionally fit than their younger peers. Thus, their functional capacities may limit their activity patterns to use destinations and functional features (e.g. sidewalks, benches) in the close vicinity of their residence. Area aesthetics were particularly important for larger buffer sizes. Elderly may only go for an extended walk when the environment is pleasant (aesthetically appealing) to walk through. Whereas other studies found that a buffer of 1600 meters was important for associations between the built environment and PA among elderly [19,20], our study did not corroborate this observation. This may be due to a distance of 1600 meters being too far for older persons to walk regardless of the area characteristics or because there was too little variation within this buffer. The larger the area in which the environment is measured, the more likely that environments of individuals will become similar which may reduce the chance of finding associations with PA levels.

Frail persons lived closer to facilities and had more functional features in their residential area as compared to non-frail persons. This could be the result of a selection process, whereby frail persons decide to move closer to facilities. However, in additional analyses, no differences between frail and non-frail persons were found in prevalence of and reasons for moving to their current residence in the past 5 years. The average total time per walk for frail persons was lower as compared to non-frail persons, which may suggest that frail elderly were more bound to smaller areas around their residences as compared to non-frail elderly. Knowledge of the exact amount of PA that was practiced within specific buffers for both frail and non-frail elderly would allow for a more accurate estimation of associations between area characteristics and walking behaviour in each buffer. It is therefore suggested to take this into account in future research, e.g. by combining GPS and accelerometer measurements [21].

Recently, differences were found in walking distances between disabled and non-disabled elderly [20]. Also, stronger associations were found between area characteristics and PA levels for disabled than non-disabled elderly [22]. As frail persons are at increased risk to develop disabilities [11,12], the role of environmental characteristics for PA may become more important with increasing health complaints.

A strength of this study concerns the personal geographical space units, i.e. the walking path based buffers around participants’ residences, instead of the often used, pre-defined geographical units, for instance based on zipcodes or neighbourhood boundaries. A personal geographical space unit provides more specific information on environmental characteristics to which persons are exposed as compared to a geographical unit. Furthermore, detailed qualitative and quantitative information about the residential areas of the elderly was collected by street audits. A limitation of this study was that area characteristics were collected up to 13 months after the first interviews took place. Thus, there is a possibility that the area characteristics may have changed meanwhile. To the extent that area characteristics determine walking, such changes in the environment may have resulted in an underestimation of the associations reported. The ISAR questionnaire was used to measure frailty, which overlaps in terms of measuring functional limitations and predicting the risk of adverse outcomes. Other studies used the Tilburg Frailty Index (TFI) which includes a broader set of indicators of frailty. It remains unknown however, of the TFI would have altered these associations [23].

As this study was conducted in a (middle-sized) city in The Netherlands, and the design of cities may differ across countries, it is unclear how these results also would apply for cities in other countries.

Our study has several implications. Firstly, for the appropriate linkage of environmental characteristics to walking (and other health behaviours), specific buffer sizes need to be used. It requires insight into the expected level of variation in the area, and it is important to realize that such variation may differ in different countries. We recommend to explore the variation of an characteristic prior to the analyses. Ultimately, such an approach may results in more consistent findings.

Secondly, living longer independently can be facilitated by a residential area that facilitates and inspires elderly to walk for daily activities. Neighbourhood improvements may increase levels of transportational walking among community-dwelling elderly. More research is needed to get more insight in the role of area characteristics for frail elderly.

## Conclusion

Better functional and aesthetic features, and more destinations in the residential area of community-dwelling older persons were associated with more transportational walking. The importance of area characteristics for transportational walking differed by size of the environmental area, but not by frailty level. Increasing functional features and the number of destinations within the area close by elderly’s residences (up to 400 and 800 meters respectively), and improving the aesthetics of a larger area up to 1200 meters, could increase their levels of transportational walking. Subsequent studies are needed to investigate whether this also results in living longer independently.

## Methods

### Design

The Elderly And their Neighborhood (ELANE) study was conducted in 2011–2012, with the aim to investigate associations between area characteristics and PA, independent living and quality of life in two samples: dismissed hospitalized older persons who participated in the Prevention and Reactivation Care Program [24], and a sample of randomly selected community-dwelling older persons. In this study we focussed on the random sample. In 2011, a sample of 2017 inhabitants of the city of Spijkenisse - a middle-sized town of about 73,000 inhabitants in the Rotterdam area, The Netherlands- of 65 years and older was randomly drawn from the municipal register of Spijkenisse. All persons included in online phone number registries (n = 1190) were sent an invitation letter and subsequently phoned for an interview appointment. In total, 1040 persons answered the phone within 5 attempts. Participants had to be non-institutionalized, not bedridden, not wheelchair or scooter-bounded, and fluent in Dutch (68 persons were excluded). Of the 972 persons eligible for inclusion, 430 were willing to participate (response 44.2%). Interviews at home were carried out between September 2011 and July 2012; winter months in between were excluded to avoid seasonal variation in PA. The study was approved by the institutional medical ethics committee (Medisch Ethische Toetsings Commissie Erasmus MC [METC]).

### Consent

Informed consent was obtained from the participants for the publication of this report and any accompanying images.

### Subjects

Of the 430 participants, 408 persons were eligible for analyses since 22 persons were excluded from analyses due to incomplete data on frailty level (n = 11), walking time (n = 4), and area characteristics (n = 7).

### Measures

#### Transportational walking

Transportational walking included grocery shopping and visiting family and friends. Total transportational walking in the past two weeks was calculated based on the answers to two questions from the Physical Activity Questionnaire in the LASA study (LAPAQ), a valid and reliable instrument to measure PA among older persons [25,26]: ‘On how many days did you walk for transport in the past two weeks?’, and ‘How long did you walk on average per day?’. We calculated total time spent on transportational walking in minutes in the past two weeks by multiplying both answers. Total transportational walking time was log-transformed, because 15% of the participants reported a walking time of zero minutes in the past two weeks. To meaningfully interpret the results, values were retransformed after the statistical analysis into minutes spent on transportational walking in the past two weeks.

#### Frailty

Frailty level was defined based on four questions measured by a short version of the ISAR (Identification of Seniors at risk of functional loss) which has proven to have sufficient validity [26-28]. Scores ranges from 0 to 5 based on the following questions: ‘Do you need assistance for instrumental activities of daily living (IADL) (e.g. assistance in housekeeping, preparing meals, shopping) on a regular basis?’ (yes = ‘1’, no = ‘0’), ‘Do you need assistance for travelling?’ (yes = ‘1’, no = ‘0’), ‘Do you use a walking device (e.g. a cane, walking frame, crutches, etc.)?’ (yes = ‘2’, no = ‘0’), and ‘Did you pursue education after the age of 14?’ (no = ‘1’, yes = ‘0’). Persons with a score of 2 or higher were defined to be frail.

#### Residential area characteristics

Around each participant’s residence, walking path network buffers were created. Starting from the nearest starting point of streets to the participants’ residence on the street network, all walking routes up to 400, 800, 1200, and 1600 meters were traced in every direction. In this way, four buffers were created using ArcGIS.

Information about area characteristics was retrieved from street audits. Between June and October 2012, we audited 88.8% (n = 918) of all streets in Spijkenisse, 214 additional street segments (as part of 143 streets), 8 parks, and 357 walking paths as identified by Google maps. When the physical lay-out of one part of a street was clearly different from other part(s) of the same street (e.g. big differences in aesthetics), it was split in two or more segments, which were audited separately. The audit instrument consisted of 41 items (Additional file 1), and inter-rater reliability was good (Cohens kappa = 0.71-0.88, p < .001). The audit was conducted by three raters (one rater per street). Since the inter-rater reliability was sufficient, all other streets were rated by one rater for practical reasons.

Separate items were taken together in overall variables for aesthetics, functional features, safety, and the presence of destinations, as suggested by the framework of Pikora et al. (2003) [29]. Scores for aesthetics were based on the following 11 items: absence of dog waste, graffiti, and litter, presence of trees, gardens, other green, water, and parks, and maintenance of the streets, sidewalks, and benches (maximum score of ‘2’ per item; total range 0–22). Functional features scores were based on 7 items: presence of a sidewalk of at least 2 meters wide at the left and right side, presence of flat curbs, benches, and waste bins, absence of sidewalk obstacles, and flatness of walking surface (i.e. paths where no cars are allowed) (maximum score of ‘2’ per item; total range 0–14). Safety scores were calculated based on the presence of crossings, speed-limiters, sufficient lighting, supervision (i.e. persons on streets are clearly visible), houses (with ground-level and without ground-level), bicycle lanes, and traffic speed limits (maximum score of ‘2’ per item; total range 0–16). The number of destinations per street was calculated based on the presence of the following 15 destinations: bus stop, supermarket, bakery, vegetable store, butcher, other shops, shopping center, hairdresser, café, ATM, sport facility, community-center, pharmacy, letterbox, and nursing home with scoring 1 per item in case one or more of that specific destination was present (maximum score of ‘1’ per item; total range 0–15). A maximum score per item means that an item contributes positively to either the sum score of aesthetics, functional features, safety, or destinations. For example, a score of ‘2’ on dog waste represents the absence of dog waste (‘1’ = little dog waste, ‘0’ = much dog waste); a score ‘1’ on supermarket represents the presence of a supermarket (‘0’ = no supermarket) (see Additional file 1).

Because the number of streets differed between buffers of different sizes and between participants, the scores for aesthetics of all audited streets within a certain buffer were summed and divided by the total number of audited streets in that buffer, resulting in an average street score for aesthetics for each buffer. The same was done for functional features and safety. For destinations, we summed the number of destinations of all the streets in each buffer.

### Statistical analyses

Initial descriptive analyses included chi-square tests and t-tests to explore sex and age differences between the participants and non-participants and between frail and non-frail persons in terms of demographics, walking, and area characteristics. Pearson correlations were calculated between the scores on aesthetics, functional features, safety, and destinations for each buffer. Finally, for each buffer a multivariate linear regression analysis was performed to test associations between area characteristics and total walking time. Adjustments were made for age, sex, and frailty. In addition, interaction effects between frailty level and area characteristics on walking time were tested. After the log transformation of walking time, residuals of the linear regression did not completely show a normal distribution, which limited the ability to calculate confidence intervals. Therefore the analyses were bootstrapped. P-values were considered significant if below 0.05. Analyses were performed using SPSS 20.0.

## Competing interest

The authors declare that they have no competing interests.

## Authors’ contribution

AE carried out the study and drafted the manuscript. All authors read and approved the final manuscript.

## Supplementary Material

Additional file 1 Street audit. Click here for file
