# Supplementary material for: Characteristics of residential areas and transportational walking among frail and non-frail Dutch elderly: does the size of the area matter?
Source: Int J Health Geogr. 2014 Mar 4;13:7. doi: 10.1186/1476-072X-13-7 (PMC4015736; doi:10.1186/1476-072X-13-7)
Supplement: Additional file 1 — Street audit. [file 1476-072X-13-7-S1.docx]

**Additional file 1.** Street audit

| **Street characteristic** | | **Score** | | | | |
| --- | --- | --- | --- | --- | --- | --- |
|  |  | **0** | | **1** | | **2** |
| Aesthetics | Litter | many | | little | | none |
|  | Dog waste | many | | little | | none |
|  | Graffiti | many | | few | | none |
|  | Park | no | |  | | yes |
|  | Maintainence benches | insufficient / n.a. | | reasonable | | sufficient |
|  | Maintainance sidewalk(s) | insufficient / n.a. | | reasonable | | sufficient |
|  | Maintainance street | insufficient | | reasonable | | sufficient |
|  | Trees | none | | few | | many |
|  | Gardens | none | | few | | many |
|  | Other green | no | | partly | | mainly |
|  | Water | no | | partly | | mainly |
| Functional | Sidewalk side 1 | absent | | yes, < 2 meters | | yes, ≥ 2 meters |
|  | Sidewalk side 2 | absent | | yes, < 2 meters | | yes, ≥ 2 meters |
|  | Obstacles sidewalk(s) | many / n.a. | | few | | none |
|  | Flatness walking surface | insufficient | | reasonable | | sufficient |
|  | Flatness curbs | insufficient / n.a. | | reasonable | | sufficient |
|  | Benches | none | | one | | more than one |
|  | Wastebin(s) | none | | one | | more than one |
| Safety | Crossings | no | | yes, without traffic light(s) | | yes, (also) with traffic lights |
|  | Speed limiters | none | | yes, one | | yes ,more than one |
|  | Lightning | insufficient | | reasonable | | sufficient |
|  | Supervision | insufficient | | reasonable | | sufficient |
|  | Land-based houses | none | | few | | many |
|  | Not land-based houses | none | | few | | many |
|  | Bicycle lane(s) | no | | yes, not seperated from carlane | | yes, seperated from carlane |
|  | Traffic speed limit^a^ | walkingpath |  | 15km road |  | 50km road |
|  |  | **Score** | | | | |
|  |  | **0** | | **0.5** | | **1** |
| Destinations | ATM | no | |  | | yes |
|  | Letterbox | no | |  | | yes |
|  | Busstop | no | | one | | more than one |
|  | Supermarket | no | |  | | yes |
|  | Bakery | no | |  | | yes |
|  | Vegetable store | no | |  | | yes |
|  | Butcher | no | |  | | yes |
|  | Other shops | no | |  | | yes |
|  | Shopping center | no | |  | | yes |
|  | Hairdresser | no | |  | | yes |
|  | Café | no | |  | | yes |
|  | Nursing home | no | |  | | yes |
|  | Pharmacy | no | |  | | yes |
|  | Community center | no | |  | | yes |
|  | Sport facility | no | |  | | yes |

^a^A combined walking/cycle path scored 0.5; a 30 km road scored 1.5
